# AI-Based Support System for Monitoring the Quality of a Product within Industry 4.0 Paradigm

**DOI:** 10.3390/s22218107

**Published:** 2022-10-22

**Authors:** Izabela Rojek, Ewa Dostatni, Jakub Kopowski, Marek Macko, Dariusz Mikołajewski

**Affiliations:** 1 Institute of Computer Science, Kazimierz Wielki University, 85-064 Bydgoszcz, Poland; 2 Faculty of Mechanical Engineering, Poznan University of Technology, 60-965 Poznan, Poland; 3 Faculty of Mechatronics, Kazimierz Wielki University, 85-064 Bydgoszcz, Poland

**Keywords:** artificial intelligence, Industrial Internet of Things, process monitoring, production optimization, 3D printing, medical devices, exoskeleton

## Abstract

Three-dimensional (3D) printing, also known as additive manufacturing (AM), has already shown its potential in the fourth technological revolution (Industry 4.0), demonstrating remarkable applications in manufacturing, including of medical devices. The aim of this publication is to present the novel concept of support by artificial intelligence (AI) for quality control of AM of medical devices made of polymeric materials, based on the example of our own elbow exoskeleton. The methodology of the above-mentioned inspection process differs depending on the intended application of 3D printing as well as 3D scanning or reverse engineering. The use of artificial intelligence increases the versatility of this process, allowing it to be adapted to specific needs. This brings not only innovative scientific and technological solutions, but also a significant economic and social impact through faster operation, greater efficiency, and cost savings. The article also indicates the limitations and directions for the further development of the proposed solution.

## 1. Introduction

Three-dimensional (3D) printing, also known as additive manufacturing (AM), has already shown its potential in the fourth technological revolution (Industry 4.0), demonstrating remarkable applications in manufacturing, including of medical devices. Three-dimensional printing offers new methods of processing and joining various materials, particularly plastics, often with specific mechanical properties (increased flexibility), including resistance to body fluids and being antibacterial or biodegradable. This encourages us to explore the development of medical devices covered by the Medical Devices Regulation (MDR) directive or the ISO 13485 standard. Compliance with all regulations requires not only precise machining with narrow tolerance (due to the requirements of therapy and adjustment to the patient), but also maintaining the required properties (material, mechanical, sometimes contradictory) throughout the entire life cycle of the product. Therefore, the production process must be closely monitored, and technical control must be carried out at every stage of the product life cycle. Multi-stage mass production of personalized products is possible under the Industry 4.0 paradigm, especially with additive printing methods (3D printing). This approach makes it easier to obtain advanced, dedicated material properties, which are easy to personalize within the product family [1,2,3]. To facilitate this, it is necessary not only to process data obtained from the Internet of Things sensor network in real time, but also to simulate, infer, and predict from such obtained data using artificial intelligence methods and techniques [4,5], including virtual twins [6]. In a broader context, this allows not only material optimization [6], but also cost, time efficiency, and product quality optimization [7]. In times characterized by an energy crisis, increases in raw material prices, and the simultaneous need for sustainable development, intelligent training optimization, including data-driven approaches (such as machine learning—ML), becomes of particular importance, both economically and socially [8].

The Industry 4.0 paradigm introduced robotization, automation, and computerization, among other things, so that the modeling and simulation of production processes in real time or close to real time would allow the most efficient and cost-effective methods of testing, selecting, and using (preferably waste-free, with recycling) technologically advanced materials as part of the modern production processes [9]. A production plant that uses, for example, advanced reverse engineering processes (3D scanning—adaptation—3D printing) should therefore provide massively personalized products with a controlled high quality [10,11].

In a broad context, it should be emphasized that our research goes beyond computer science, mechanical engineering, and materials engineering, influencing medical sciences and health sciences (physiotherapy, rehabilitation), as well as humanities and social sciences (e.g., psychology). It is important not only scientifically, but also economically and socially, and our results may have a multidirectional impact.

The analysis of the current state of the research field showed that there is a research gap in the area of the selection of material and technological parameters for the needs of individually tailored 3D-printed exoskeletons [12,13]. In order to determine the state of the art, we performed a literature review of five major bibliographic databases based on specified keywords in English: artificial intelligence, Industrial Internet of Things (IIoT), process monitoring, production optimization, 3D printing, medical device, and related items. For the above-mentioned keyword chain, we did not find any publications, so we consider our article to be pioneering. In contrast, of the above-mentioned keywords, the majority of papers included the combination of the keywords ‘artificial intelligence’ and ‘industrial internet of things’, a total of 1230. The results of the review form the starting point of this article.

The aim of this publication is to present a novel concept of support by artificial intelligence (AI) for the quality control of additive manufacturing of medical devices made of polymeric materials, based on the example of our own elbow exoskeleton.

While the work to date mainly concerns semi-serialized 3D printing of products, our research concerns 3D printing of personalized medical devices (exoskeletons). In our case, the differences between successive products are much greater, as each product involves individual (patient-specific) modification of the overall design. The amount of modification varies according to the dimensions and personal functional capabilities of each patient. Hence, our case represents a rarity and a novelty, capable of becoming the beginning of the field of research on personalized medical devices that comply with the Medical Devices Regulations and the ISO 13485 standard.

**Figure 1:**
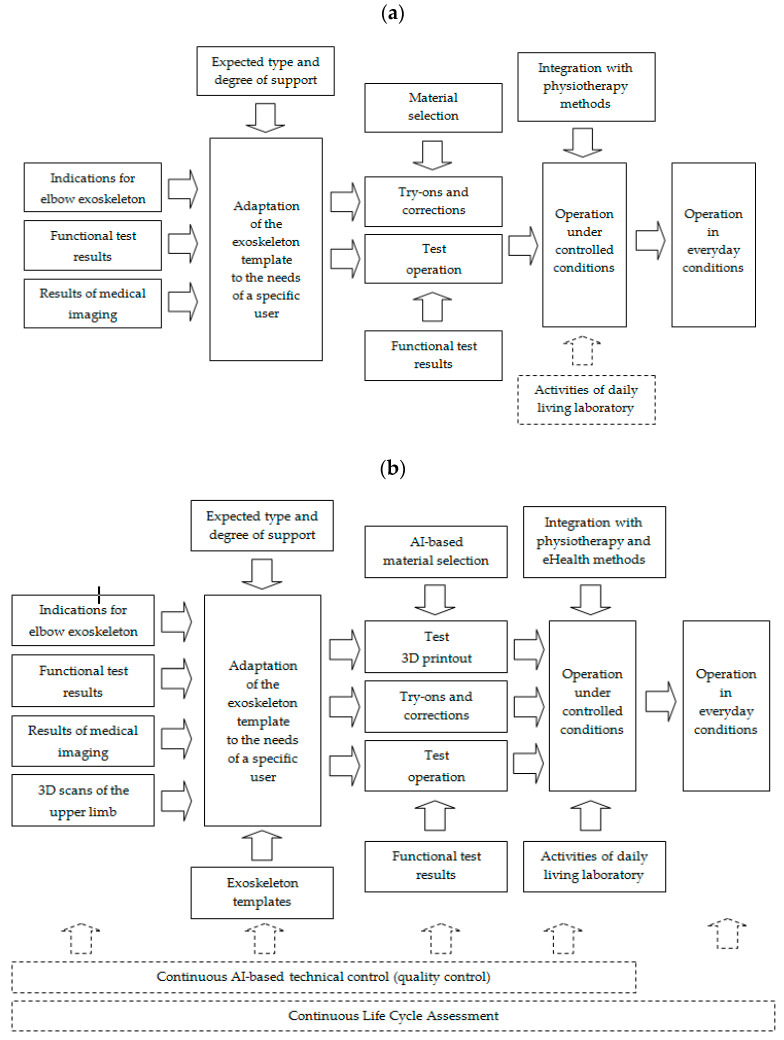
Approach to elbow exoskeleton production: (**a**) traditional, (**b**) novel AI-based within Industry 4.0 paradigm.

The comparison between the current situation and the proposed solution covered by this study is shown in Figure 1. The proposed concept includes not only an increase in the use of AI for the selection of materials and exoskeleton fabrication technology, but also a comprehensive technical control and product life cycle analysis (LCA).

The novel approach to manufacturing the elbow exoskeleton (based on AI within the Industry 4.0 paradigm) introduces several key elements to increase control and efficiency throughout the process:

- Three-dimensional scans of the upper limb allowing the personalization of the product and a better fit to the patient’s current condition.
- Digital templates of the exoskeleton in 3D, making it easier to process and speeding up the preparation of the finished prototype.
- Faster fitting and adjustments.
- Faster implementation of observations from the physiotherapy process and from the practical use of the exoskeleton in the activities of daily living (ADL) laboratory.
- Technical control at each stage of design and production (analysis of the accuracy of manufacture and achievement of the required functional properties of the product), both on the basis of data from design software and 3D printers and from additional sensors and surveys (e.g., satisfaction surveys of the patient and the physiotherapist who treats him or her), while keeping in mind that a product such as an exoskeleton serves the patient and must be perceived by the patient as useful and increasing his/her health-related quality of life (HRQoL).
- LCA based on data from the design, production (including waste reuse), and the process of tracking the fate of each exoskeleton component (replaced, repaired, recycled) to achieve maximum material utilization.
- Collecting and using data from previous similar products to aggregate into the knowledge on the most cost-effective, energy-efficient, and sustainable design and production methods.

We must take into account that cost accounting or production profit alone will not be the only indicator of the profitability of production in the coming decade, and that environmental or other factors falling within the EU’s horizontal policies will be among the important values. For the aforementioned reasons, all processes will be subject to the proposed modifications, and non-economic factors will play an increasingly important role (e.g., the role of fishing in the emergence of the Great Pacific Garbage Patch and the proposed ways to reduce it until it is completely eradicated). For the aforementioned reasons, the work is pioneering a huge market for medical devices, assessed at USD 488 billion in 2021, with a compound annual growth rate (CAGR) of 5.5% until 2029. The growing number of medical devices used and their packaging necessitate a wider implementation of the proposed approach in the coming years.

## 2. Materials and Methods

The methodology of the above-mentioned inspection process differs depending on the intended application of 3D printing, 3D scanning, or reverse engineering.

The novelty of the proposed approach and the associated research project lies in the application of AI to optimize the fabrication of a personalized medical device (exoskeleton), filling an observed research gap, and the results provide evidence of the validity of our prototype approach, not previously encountered in the literature. The computational solutions used, including in an environment such as MATLAB, were developed for the prototype and will be replaced in the target solution by more advanced target solutions, perhaps specifically designed for such applications. The creative application of AI-optimized 3D printing is still in its infancy but is an important area of Industry 4.0 supervised using the IIoT.

### 2.1. Materials

The described invention develops medical technologies in non-invasive therapy and rehabilitation and care in the form of an exoskeleton, a device placed on the upper limb, supporting the user’s movement in a passive way (facilitating movement through partial relief and supporting movement with rubber bands, springs) or in an active manner (facilitating movement by means of actuators, etc.). The elbow joint is the most complex human joint. The occurrence of a functional deficit, weakness, or fatigue in the upper limbs, particularly the elbow joint, is a global problem: annually, in Poland alone, it has reached 400–800,000 from strokes, which are one of the causes of deficits of this type. In addition, a significant group of patients may be elderly people (6–10 million Poles), who experience a similar negative deterioration in the function of the upper limb due to neurodegenerative changes in the aging process. With the above reasons, any deficit in this area reduces the health-related quality of life, and for the above-mentioned elbow joint deficits, there are no other alternative solutions apart from the proposed exoskeleton.

The solution implements the concept of personalized therapy, combining mass production on the basis of a template and matching product features to the requirements of the therapy of a specific patient, which is in line with the Industry 4.0 paradigm. Three-dimensional scanning allows one to record the characteristics of the structure of the upper limb in the form of digital files, and the combination of 3D scanning technology and 3D printing in the form of reverse engineering allows the creation of a relatively cheap (with adaptation to a specific user) digital design of an exoskeleton with a complex internal and external structure based on a physical counterpart (including anatomical—measurements made in the patient). Three-dimensional printing allows one to create an exoskeleton from digital files, with parameters selected individually for each user (dimensions, strength, flexibility, weight, support force, etc.). It also reduces the number of necessary patient visits and reduces the waiting time for the finished exoskeleton.

Advantages over competitive solutions include the following.

1. Quality advantages:
  - Individual adjustment and execution using 3D scanning and 3D printing methods;
  - Supporting the functions of the elbow joint;
  - Immediate improvement of functions (reaching, interaction with objects); and
  - Shaping the improvement of function over a longer period of time.
2. Technological advantages:
  - Possibility of everyday use at home; and
  - Gradual adaptation (including through the replacement of elements) to changes in health.
3. Ecological advantages:
  - Solution that changes with the patient (with adjustable or replaceable elements, ecological advantage—no need to replace the entire device);
  - Three-dimensional printing from organic and recycled materials; and
  - The ability to only replace individual parts as the degree and type of deficit changes (including healing, worsening).
4. Cost advantages:
  - Domestic production; and
  - Lower costs, service, and helpdesk.
5. Price advantage: on-site availability (including testing and scanning).

**Figure 2:**
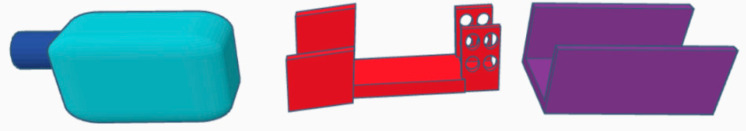
Parts of the exoskeleton for the elbow joint.

**Figure 3:**
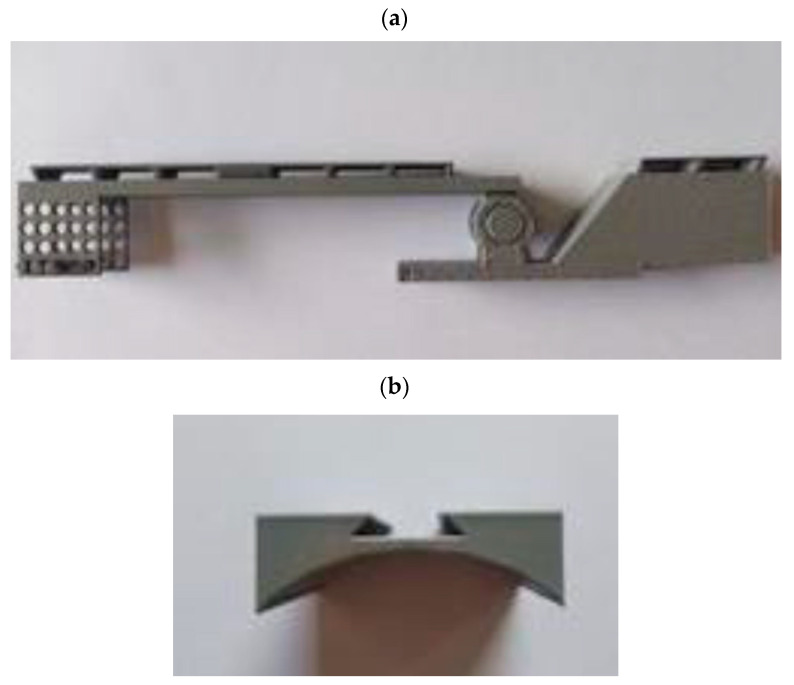
Strength test samples—parts of the exoskeleton for the elbow joint: (**a**) lever (five samples), (**b**) cap (four samples).

The described invention develops medical technologies in non-invasive therapy, rehabilitation, and care. The primary goal is to support the patient’s rehabilitation and independent functioning in normal home conditions. The aim is also to introduce a new product to the market that offers a new functionality on a national scale: a passive exoskeleton (support based on elastic elements) and an active exoskeleton (support based on actuators) for people with a function deficit and/or weakened muscle strength in the area of the elbow joint (Figure 2). The therapeutic effect is immediate, but achieving full proficiency in the use of the above-mentioned exoskeleton by the user requires functional training combined with fine-tuning of the exoskeleton mechanism. The supply of an exoskeleton may apply to one or both upper limbs, and their functional deficits and the degree of support offered by the exoskeleton may not be the same. The solution also takes into account that the functional capacity of the hand is subject to changes during the process of self-healing and rehabilitation stimulated by rehabilitation. The safety of the elbow exoskeleton is a key value, so the results of strength testing are important for the entire design and production cycle (Figure 3). ‘Exoskeleton for the elbow joint’ is the subject of a pre-implementation grant in the years 2021–2022 as part of the ‘Innovation Incubator 4.0’ project. The target group includes patients with a functional deficit in the hand area (congenital, traumatic, neurodegenerative, etc.). The market is massive (global), as the occurrence of a functional deficit, weakness, or fatigue in the upper limbs, particularly the elbow joint, is not subject to geographical divisions.

### 2.2. Methods

**Figure 4:**
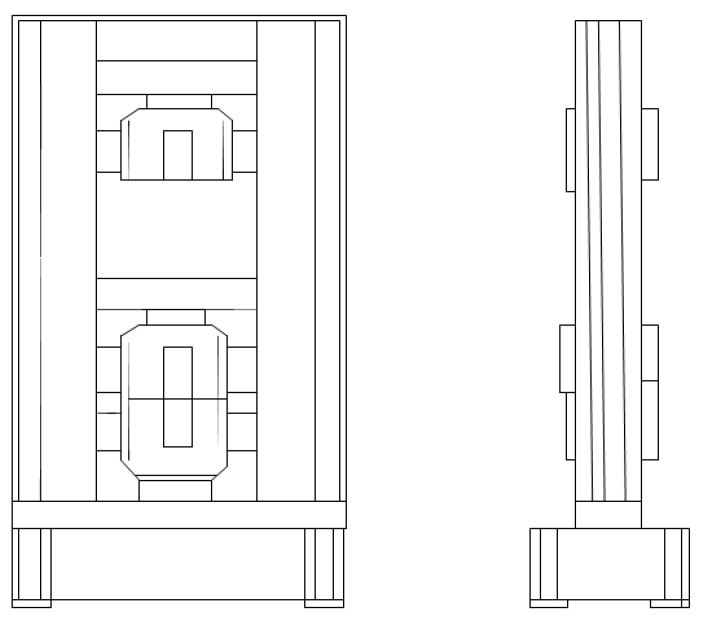
LABTest 6.100 device (front view and side view).

A tensile strength test was performed using a universal machine strength LABTest 6.100 (LaborTech, Opava, Czech Republic, machine 1 accuracy class, Figure 4), using the following test conditions and parameters:

- Test temperature: 21 °C;
- Test speed: 5 mm per min; and
- The samples were mounted on the machine each time using twisted jaws.

A compression test was performed using the same machine using the following conditions:

- Test temperature 23.4 °C; and
- Compression speed: 2 mm per min.

### 2.3. Statistical Analysis

The MS Excel/Office 365 spreadsheet (Microsoft Corporation, Redmond, WA, USA) was used for data archiving. Design data sets from a 3D printer, material properties, and calculation/simulation results were statistically analyzed using Statistica 13 (manufacturer: StatSoft Inc., Tulsa, OK, USA). Where possible, we have represented the results using statistics: for data with a normal distribution, using the mean and standard deviation (SD); for data with a distribution different from the normal distribution, using the median, minimum, and maximum values, and the lower and upper quartiles. We checked the normality of the distribution using the Shapiro–Wilk test.

### 2.4. Computational Analysis

The data for computational analyses were taken from the elbow exoskeleton design, printer software, and selected according to the experience and knowledge of the system designers. All simulations were carried out with the use of MATLAB software + toolboxes Neural Networks (MathWorks, Natick, MA, USA).

**Figure 5:**
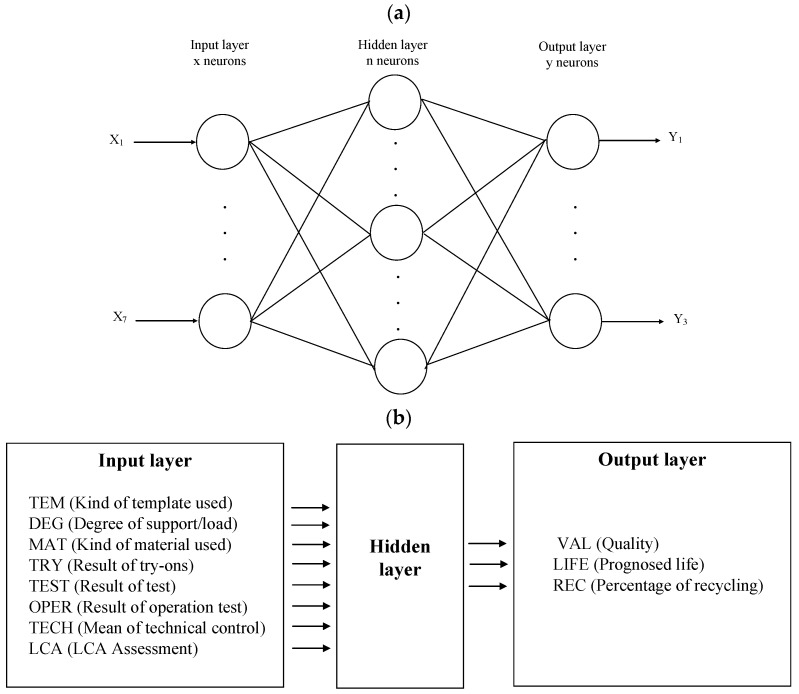
Traditional ANN structure used in the study: (**a**) general structure, (**b**) inputs and outputs.

The proposed structure of the artificial neural network is a traditional MLP, i.e., a multi-layer perceptron network (Figure 5).

The MLP network was chosen based on the authors’ knowledge, previous research, and experience. The MLP network has a three-layer structure: an input layer, a hidden layer, and an output layer (Figure 5). Eight neurons were placed in the input layer, each corresponding to one parameter value from Figure 1b. Three neurons were placed in the output layer, each of which corresponded to one of the three parameters corresponding to product quality, predicted durability, and recyclability. The number of neurons in the hidden layer was first estimated based on the authors’ previous knowledge and experience, followed by an experimental analysis of the most likely MLP network structures. The MSE values, the transition functions, and the results for the most probable MLP networks are presented in Section 3–Results. It follows, as discussed later in the paper, that the best results were achieved with an MLP 8-10-3 network, which is an MLP network with 8 neurons in the input layer, 10 neurons in the hidden layer, and 3 neurons in the output layer. Values of TEM, DEG, and MAT were assessed by the experiment described later in this article. Values of TRY, TEST, OPER, and TECH were submitted by sensors or human action (e.g., manually by physiotherapist) after finishing subsequent phases of the designing process from Figure 1b. LCA assessment was calculated using a simple tool (OpenLCA, https://www.openlca.org, accessed on 15 June 2022), but many diverse tools may be used here, beginning with LCA Calculator, Eco Indicator 99, Eco Scan 3.0, or Eco-IT through OpenLCA to advanced SimaPro, GaBior Umberto analysis. OpenLCA has been used in many studies thus far (https://www.openlca.org/openlca-publications-research/ (accessed on 15 June 2022)).

## 3. Results

The use of artificial intelligence in the form of traditional or deep neural networks increases the versatility of this process, allowing it to be adapted to specific needs. This brings not only innovative scientific and technological solutions, but also a significant economic and social impact through faster operation, greater efficiency, and cost savings.

Optimization of parameters related to the 3D printing process is a key problem that these AI methods solve. In order to ascertain the quality of the optimization, we assisted it by a genetic algorithm (GA). In this case, with the approach to control material selection and component quality, the use of an ANN in combination with a genetic algorithm is innovative and allows us, for example, to maximize material strength with given materials.

### 3.1. Results of the Experimental Research

**Figure 6:**
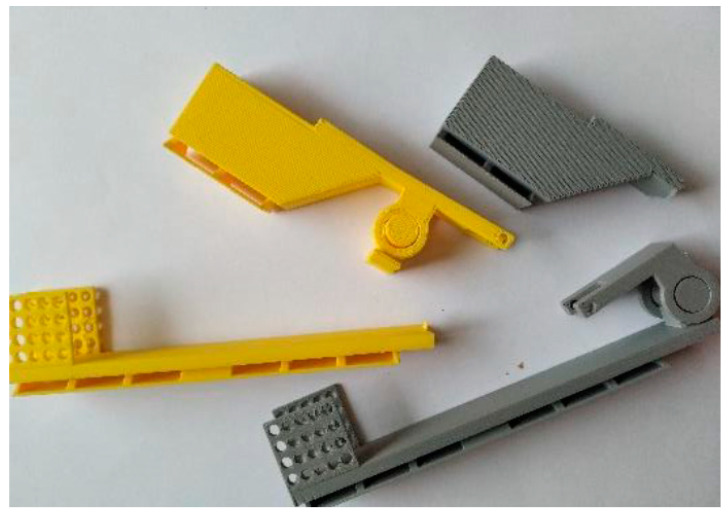
Selected samples (lever) after the test.

We conducted experimental research to explore values of TEM, DEG, and MAT parameters for AI. Selected samples of the exoskeleton were tested toward the measurement of compression forces and tensile forces (Figure 6).

**Figure 7:**
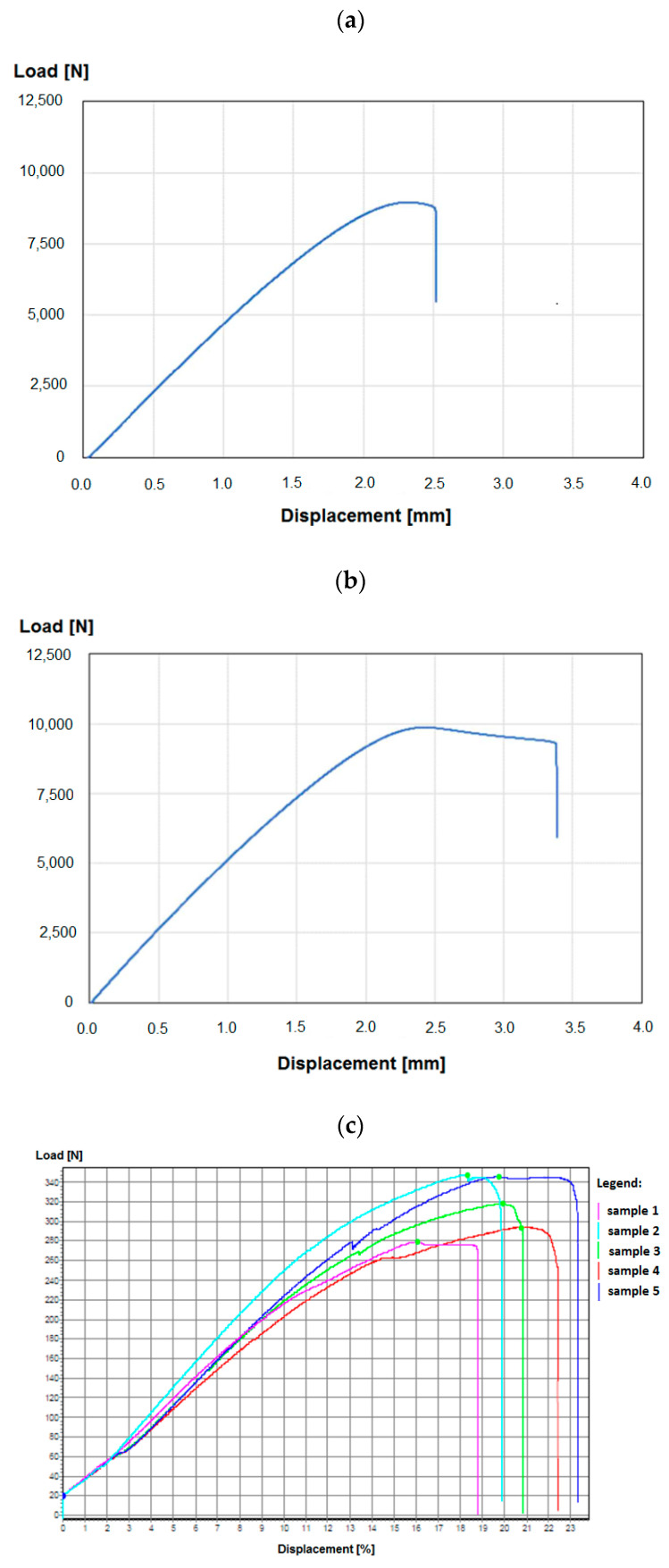
Selected tensile strength test results: (**a**) lever, (**b**) cap, (**c**) tensile strength of the cap—comparison of results for individual samples.

The main results of the test are shown in Table 1 and Figure 7. The actual results from the strength tests shown in Table 1 and Figure 7a–c indicate which components of the exoskeleton, working under load, are crucial to the strength of the product and, as a result, its safe use and durability (life span). The analysis of the above-mentioned parameters in different cases allows the material and the features of the manufacturing process to be appropriately selected (optimized) at the planning stage, and then reflected in the form of a simple virtual twin (the digital equivalent of the product or its parts), which is gradually extended. Also crucial for further analysis is the fact that we gain access to continuous characteristics and not just single parameter values.

### 3.2. Results of the Computational Analysis

The best ANN for this task was MLP 8-10-13 with sigmoid transfer functions (Table 2). Other transfer functions tested (threshold, linear, Gaussian) and their combinations gave worse results.

**Figure 8:**
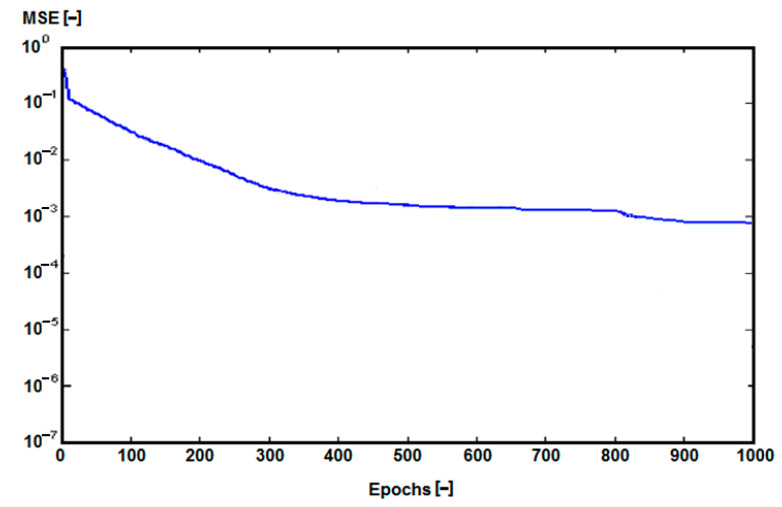
Values of MSE during learning for ANN (MLP 8-10-3).

Scores achieved by MLP 8-10-13 were the best in terms of quality (learning) 0.9452, quality (testing) 0.9767 (Table 3), and MSE 0.01 (Table 4, Figure 8). We carefully checked almost 100 different network configurations, and in the following tables, we give the results for only the best of them.

The change in MSE for the aforementioned neural network structure is shown in Figure 8.

To increase reproducibility, we have shown the settings of the artificial neural network parameters and the learning algorithm (Table 5).

## 4. Discussion

Quality, durability, and prognosed life of the elements of the exoskeleton are key indicators of its safety. Moreover, the percentage of recycled parts is key to provide environmentally friendly solutions within the sustainability of healthcare products. Non-technological parameters engaged here may be crucial for the choice of the particular exoskeleton construction; thus, the proposed AI-based system may be a significant step in the proper direction, and worthy of further development.

The implications of our results against the background of the research conducted thus far can be considered broad and significant—they open up promising directions for further research.

There is no doubt that the fourth technological revolution related to the introduction of robotization, computerization, and automation has already touched all areas of production, and is even going further: the tariffs of the life cycle of products and services and the related tasks of the organization. Our research results represent significant scientific, clinical, social, and economic progress in the areas of:

- AI-supported decision-making processes within production and logistic support;
- Technical support for clinical practice (including eHealth and Clinic 4.0);
- Interdisciplinary research in teams combining knowledge and experience in many fields of science and clinical practice;
- Technical support for the process of therapy and care for people with deficits in the elbow joint; and
- Personalized therapy (patient-tailored therapy), individually tailored to the patient’s needs and changing with their health condition, goals, and needs.

Most of the current research efforts are focused on the development of 3D-printed medical systems, presenting various scenarios for installing 3D printers in industry, but also in hospitals, rehabilitation centers, pharmacies, and even in patients’ homes. Research to date has focused on identifying and analyzing a number of parameters/factors that need to be considered to integrate 3D printing into the current and future healthcare system, including telemedicine services, in the absence of a sufficient number of medical professionals. This applies in particular to the issues of legal and ethical regulations (including the so-called ethical AI), but also shortages of materials and drugs, and the need to ensure a sufficiently high quality of the product. The acceptability of the above-mentioned directions of the healthcare system development by medical specialists, patients, and their families is crucial to include in the research spectrum of possible future scenarios for the implementation of 3D printing in healthcare and to discuss problems that must be foreseen and solved before they even appear [9]. Budzik et al. [10] proposed a comprehensive control system for the additive manufacturing process for Industry 4.0. The results show that the PolyJet method is the most accurate and the MEM method is the least accurate. Metrological conclusions are also important. The choice of materials and 3D printing technology, as well as the selection of measurement methods, should take into account the specificity and purpose of the model as well as the economic aspects of its implementation. It is worth considering the complexity of the system and its required accuracy. Even in the case of medical devices, not all products require high accuracy and durability [10]. Turek et al. [12] proposed the application of anatomical models and surgical templates in maxillofacial surgery—it caused an increase in accuracy with a simultaneous reduction in the time needed to develop a decision, which accelerated the patient’s rehabilitation and their return to the highest achievable functional fitness [10]. Xiong et al. [13] proposed a 3D-printed approach to mammoplasty that achieved personalized pre-operative design and patient education, shortening the operation time and avoiding harmful secondary changes [13]. Our proposal goes further: it provides individually tailored, reusable rehabilitation supplies with controlled wear, and is technologically ground-breaking as an exoskeleton for the upper limbs. The proposed AI-based improvement of the production process may be a significant novelty here, because the production of these types of products was only possible on a random basis, due to the need to repeatedly measure and adjust them, preceded by a functional diagnosis.

### 4.1. Limitations of Own Research

The limitation of our research is the number of analyzed factors and their difficult description, often expressed linguistically. The presence of imprecision and uncertainty creates the need to enrich the computational apparatus in the future with further analytical methods, particularly fuzzy logic and fractal analysis [14,15,16,17]. We are working on developing methodologies for automated surveys, including using the Internet of Things. Further publications of our research results are planned.

### 4.2. Directions for Further Research

Hence, the main direction of further research will be to supplement the proposed system based on a neural network with a hierarchical fuzzy system enabling the processing of parameters expressed linguistically and reducing the uncertainty of adjusting the result to the expectations (including medical specialists and patients). In addition, the current system proven under scientific conditions should be translated into a commercial solution, i.e., adapted to the API of specific 3D scanning and printing devices and their software (CAD, slicer). In a broader medical context, it is possible to monitor changes in the parameters of a given patient’s devices in terms of analyzing the trend of changes in their health condition and predict future results, and thus, modifications necessary for gradual introduction in a medical device (as part of the adjustment or replacement of certain parts). This will extend the product life cycle and make it more environmentally friendly. Such replacement of parts, depending on the patient’s health, may allow for an upgrade to a newer model without having to replace the entire device—nowadays, this is sometimes executed in wheelchairs. This approach should be promoted as it provides better coverage of patients’ needs at the same cost, which is of particular importance given the aging of society and the need to provide rehabilitation equipment to a greater number of elderly people.

An additional advantage of this article is the discussed planning and production of the exoskeleton on the elbow, which is not often found in the literature [18,19], and is unique in the area of 3D-printed solutions. Most contemporary research focuses on monitoring the parameters of the rehabilitation process [20,21,22,23]; thus, the methodology used is quite different (e.g., principal component analysis (PCA)) [21].

Generally, the above approach is another step towards the Factory of the Future [24], where complex and even conflicting customer requirements exceed the capabilities of traditional factories. The Industrial Internet of Things (IIoT) is making it possible to address the challenges of remote monitoring, intelligent analytics, and control of industrial processes in a mass production environment of 3D-printed individualized products [25]. Here, IIoT combines machines on the production line with smart sensors and effectors to optimize the approach needed for flexible manufacturing and industrial processes with data-driven condition monitoring, based on three main layers: sensor layer, edge layer, and centralized cloud [26].

IIoT systems produce or collect huge amounts of data with a wide range of possible uses, and the analysis, inference, and prediction systems based on them provide a new tool for discovering new knowledge and making decisions, but also for predicting future knowledge and preparing scenarios for responding to it. Enhancing the capabilities offered by technology, including from a business perspective, such as detecting trends or anomalies and automatically responding to them, is the domain of artificial intelligence, including data-driven machine learning solutions, i.e., traditional and deep neural networks [27,28].

When discussing future plans in relation to the status of the research and its limitations, we must identify the following research priorities:

- In the hardware area: expanding the number and types of sensors and effectors that can be used; and
- In the software area: expanding the system’s capabilities to include non-precision data processing, inference, trend analysis, and prediction [29,30].

The above-mentioned approach to planning the development of the system will allow us to adapt better and faster to market requirements and find potential customer groups [31,32].

## 5. Conclusions

Modern, automatically monitored production processes are currently the key resource supporting further logistic and marketing activities of companies. AI is a natural direction for the development of Industry 4.0 systems. This is due not only to the use of the IIoT (and cloud computing), but to the need to ensure the adaptability of production systems and the necessary speed of decision making in near-real time. The accuracy of the measurement (and the mapping in the system or virtual twin) and the speed of reaction can also be decisive in avoiding damage to the production system. The scope of future work includes the development of both the hardware part (new sensors, effectors) and the software part (use of fuzzy logic, multifractal analysis), which will result in a better match between the proposed solutions and the needs of the growing market.

## Figures and Tables

**Table 1:** Strength test results.

Sample (Part of Exoskeleton)	Unit	Mean ± SD ^1^
Compression test		
Lever	(MPa)	3.00 ± 0.2
	(N)	9602.5 ± 557.74
Cap	(N)	9877.4 ± 462.58
Tensile strength test		
Lever	(N)	316.99 ± 9.62
Cap	(N)	437.13 ± 13.22

^1^ SD—standard deviation.

**Table 2:** ANN network model.

NS	AH	AO
ANN	Sigmoid	Sigmoid

**Table 3:** Quality assessment of selected ANNs.

Network Name	Quality (Learning) (Mean ± SD)	Quality (Testing) (Mean ± SD)
MLP 8-8-3	0.8755 ± 0.002	0.8947 ± 0.002
MLP 8-9-3	0.9051 ± 0.001	0.9423 ± 0.002
MLP 8-10-3	0.9452 ± 0.001	0.9767 ± 0.001
MLP 8-12-3	0.9122 ± 0.001	0.9324 ± 0.001
MLP 8-15-3	0.8754 ± 0.002	0.9021 ± 0.001

**Table 4:** MSE values for used ANNs.

Network Name	MSE (Mean ± SD)
MLP 8-8-3	0.04 ± 0.01
MLP 8-9-3	0.02 ± 0.005
MLP 8-10-3	0.001 ± 0.0002
MLP 8-12-3	0.02 ± 0.005
MLP 8-15-3	0.04 ± 0.01

**Table 5:** Optimal parameter settings of the artificial neural network and the learning algorithm (MLP 8-10-3).

Name of the Parameter	Value
Model setup	
Number of layers	3
Number of neurons in hidden layer	10
Activation function	Sigmoid
Learning hyperparameters	
Number of training iterations	1000
Learning rate	Gradient descent
Momentum parameter	0.9
Learning algorithm	Multilayer perceptron
Loss function	MSE
Dropout (if used)	0.8
L1, L2 regularization (if used)	
Lambda 1	0.02
Lambda 2	0.05

## Data Availability

Not applicable.
